# Supplementary material for: Modified MRI Anonymization (De-Facing) for Improved MEG Coregistration
Source: Bioengineering (Basel). 2022 Oct 21;9(10):591. doi: 10.3390/bioengineering9100591 (PMC9598466; doi:10.3390/bioengineering9100591)
Supplement: Supplementary file 1 [file bioengineering-09-00591-s001.zip › bioengineering-1937115-supplementary.pdf]

Supplementary Material

CBU group: ratings of familiarity

The CBU group was familiar with some of the people whose images were used. Individual familiarity ratings provided by the CBU group were positively related to the identification accuracy at the single-trial level. This was confirmed in a linear mixed effects logistic regression that modelled condition, order and familiarity as fixed and subject and faces as random effects. The analysis revealed a significant linear effect of familiarity ($F(1,1474) = 3.73$, $p = 0.05$) in addition to a significant condition by order interaction ($F(6,1474) = 2.48$, $p < 0.05$).

Face identification effect

Some faces were easier to identify than others, most likely due to some idiosyncratic features in their face and head shape. An Analysis of Variance (ANOVA) confirmed a significant main effect of Face ($F(9,810$, epsilon = 0.87) = 22.2, $p < 0.001$) when collapsing across conditions, but this effect was also observed in analysis that solely focussed on the defaced condition ($F(9,810$, epsilon = 0.81) = 6.48, $p < 0.001$) see Figures S1 and S2.

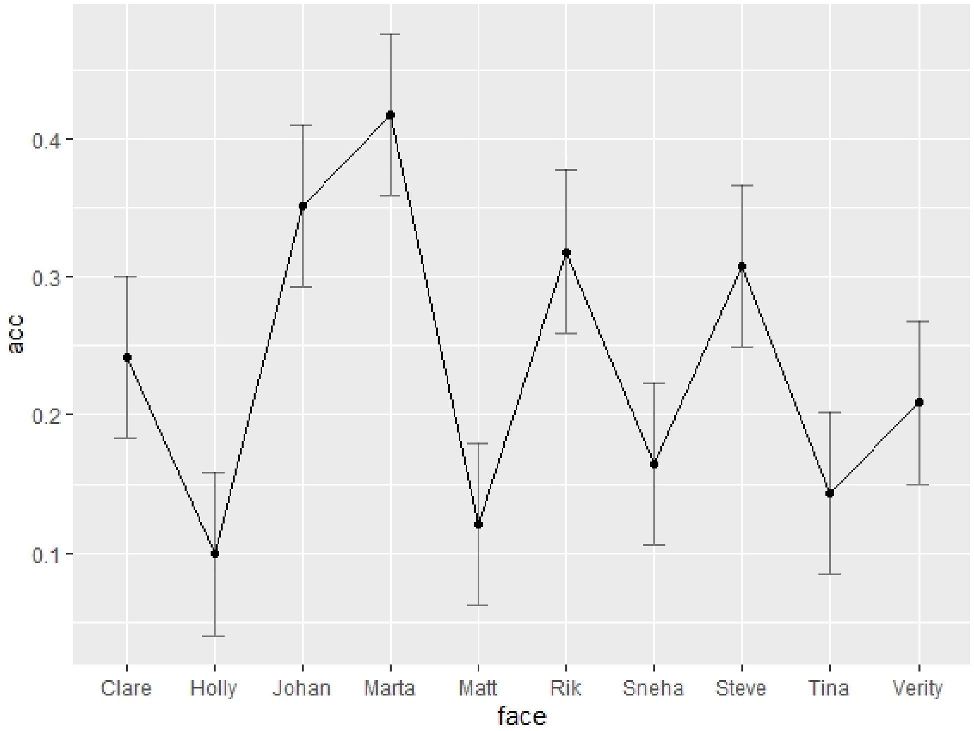

Figure S1. Accuracy in face identification was systematically better for some faces compared to others, even when considering the Defaced condition only, which is shown here collapsed across different blocks.

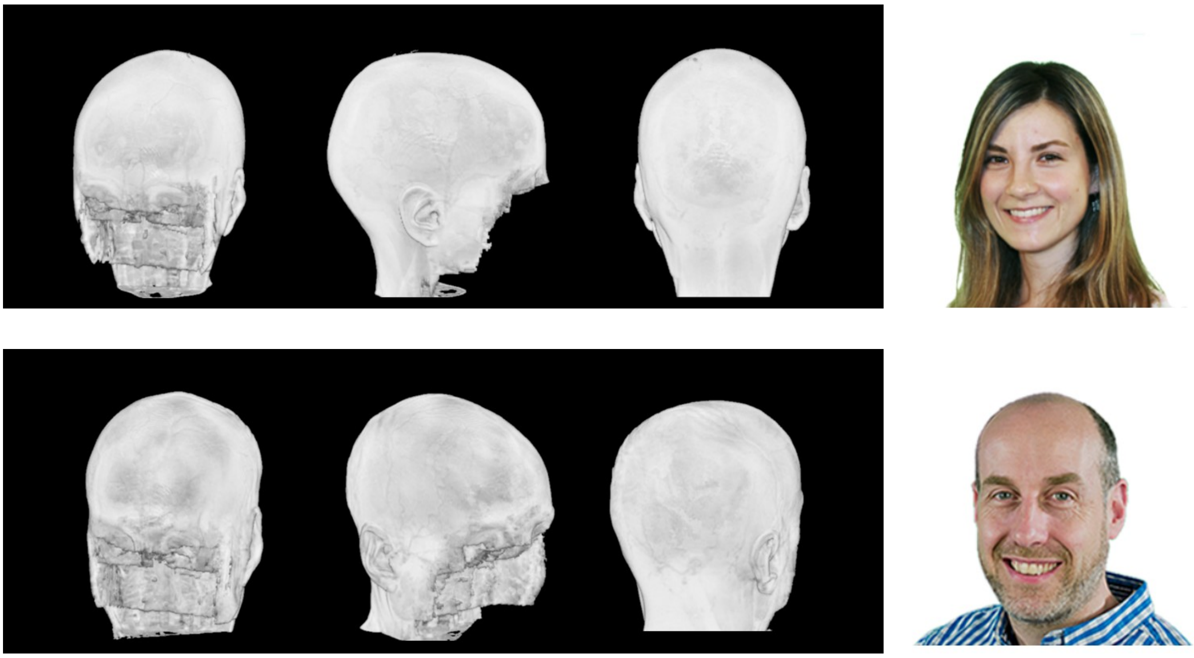

Figure S2. Example of visual detail presented in the Defacing condition relative to the target image that has been familiarised at the start of the session and was one of the 10 alternative forced choices. However, note that defacing image and target image are shown here in parallel for illustration purposes only. The alternative force choice options were displayed once the defaced image disappeared from the screen, to prevent visual matching strategies.

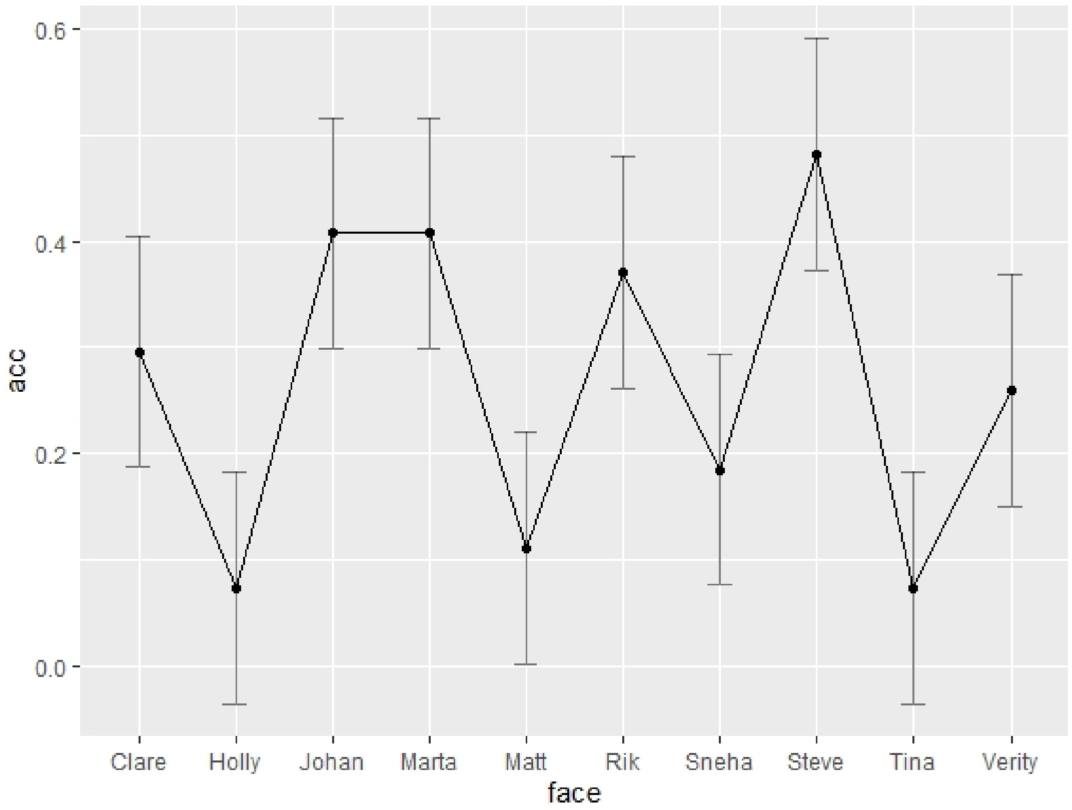

Figure S3. Accuracy in face identification was systematically better for some faces compared to others, even when considering the Defaced condition only, which is shown here for the first block only.
